# Mulberry Extract Mitigates Glucose‐Induced Oxidative Injury in Differentiated ARPE‐19 Cells by Enhancing Antioxidant Defense: Implications for Diabetic Retinopathy

**DOI:** 10.1002/fsn3.70180

**Published:** 2025-05-05

**Authors:** Pornpan Sukboon, Rianthong Phumsuay, Chadamas Promkum, Parunya Thiyajai, Monruedee Sukprasansap, Chawanphat Muangnoi

**Affiliations:** ^1^ Community Nutrition Unit, Institute of Nutrition Mahidol University Nakhon Pathom Thailand; ^2^ Cell and Animal Model Unit, Institute of Nutrition Mahidol University Nakhon Pathom Thailand; ^3^ Food Toxicology Unit, Institute of Nutrition Mahidol University Nakhon Pathom Thailand; ^4^ Food Chemistry Unit, Institute of Nutrition Mahidol University Nakhon Pathom Thailand

**Keywords:** diabetic retinopathy, differentiated ARPE‐19 cells, intracellular antioxidant, mulberry, oxidative injury

## Abstract

Diabetic retinopathy, or DR, is an eye disease that causes globally impaired vision and vision loss among working‐age adults. The progression of DR is related to persistent metabolic disturbances resulting from hyperglycemia. In diabetic patients, retinal cells, including RPE, are continuously exposed to high glucose conditions, which trigger oxidative stress and injury and finally lead to DR. Mulberry is rich in phytochemicals that offer various health benefits and is a significant source of anthocyanins, known for their antioxidant properties. Our research aimed to assess the antioxidant effects and underlying mechanisms of mulberry extract (ME) on high glucose‐induced oxidative injury in human retinal (differentiated ARPE–19) cells. Differentiated ARPE–19 was treated with ME at 5, 50, and 500 μg/mL for 24 h, followed by incubating with 25 mM glucose for 48 h. From the HPLC analysis, ME contained anthocyanin compounds, including C3G and C3R, at 8.13 and 9.86 mg/g of dry weight, respectively. ME could prevent differentiated ARPE–19 cells from oxidative injury by mitigating ROS generation, modulating protein expression in the apoptosis markers (Bax, cytochrome *c*, Bcl–2, caspase–9, and –3), and enhancing the capacities of intracellular antioxidants by activating the critical transcription factor Nrf2 in a dose‐response relationship. Additionally, ME could boost the ability of the intracellular antioxidant system of human retina cells in normal conditions. However, this study is only a cell culture study. Thus, the health benefits of mulberry, especially eye health and vision, and preventive measures against DR should be further evaluated in animals and humans.

## Graphical abstract

Mulberry extract protects differentiated ARPE‐19 cells from high glucose‐induced oxidative injury by reducing ROS generation, inhibiting mitochondrial apoptosis via modulation of Bax/Bcl‐2 and caspase pathways, and enhancing intracellular antioxidant defenses via Nrf2 activation.

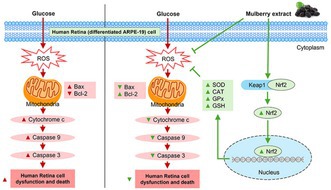

## 1 Introduction

Diabetic retinopathy, or DR, is an eye disorder that is the most common complication in diabetic mellitus patients, posing significant risks to life and health. It causes low vision and vision loss among working‐age adults globally. The development of DR is linked to prolonged metabolic disturbances resulting from chronic hyperglycemia. Sustained hyperglycemia is an initiating factor, inducing oxidative stress and chronic inflammation. This leads to circulatory system changes, damage to the retinal blood barrier (RBB), gradual retinal vascular basement membrane thickening, leakage of retinal microvessels, and edema. These lesions progress concurrently with the advancement of diabetes (Ansari et al. 2022; Duh et al. 2017). The retina, a highly specialized tissue, is crucial for protecting the neural tissues of the RBB. The human retinal pigment epithelium (RPE) cells consist of epithelial cells in a single layer, laterally connected at their apices by tight junctions (TJs) between adjacent plasma membranes. This layer lies at the interface between the choriocapillaris and the photoreceptor. RPE cells form the outer BRB, safeguarding the retina by preventing the influx of plasma components and toxic molecules (Simó et al. 2010; Wang et al. 2023).

In diabetic patients, retinal cells, including the RPE cells, are continuously exposed to high glucose conditions, leading to oxidative stress injury, cellular dysfunction, and death. Previous studies have demonstrated that injury from oxidative stress, caused by a diabetes‐induced metabolic disorder resulting from diabetes‐induced metabolic abnormalities, is the primary mechanism involved in the pathophysiology of DR (Cecilia et al. 2019; Tarr et al. 2013). In chronic hyperglycemia, the uncoupling of mitochondrial electron transport generates highly reactive molecules, including ROS, such as OH˙^−^ and O_2_˙^−^ radicals. These free radicals can activate four abnormal biochemical pathways: increased flux through the polyol pathway, enhanced intracellular production of advanced glycation end‐products (AGEs) and expression of their receptor, stimulation of the protein kinase C (PKC), and the hexosamine pathway (Antonetti et al. 2021; Yang et al. 2021). Hyperglycemia‐induced abnormal metabolism leads to the excessive generation of ROS within RPE cells, resulting in oxidative injury to macromolecules of RPE cells, including DNA, proteins, and lipids. This harmful condition also induces mitochondrial dysfunction. Consequently, oxidative stress can damage cellular structures, impair cellular functions, and lead to cell apoptosis. This apoptosis is modulated by key apoptosis proteins, including Bax (proapoptotic); Bcl–2 (anti‐apoptotic); cleaved caspase–2, –8, –9, and –10 (initiator caspases); and cleaved caspase–3, –7, and –6 (effector caspases), as well as the release of cytochrome *c* from mitochondria to the cytoplasm (Sinha et al. 2013). Furthermore, imbalances in biochemical metabolites within the diabetic retina can disrupt the secretion of various biological mediators, including cytokines/chemokines, growth factors, vasoactive agents, and adhesion molecules. These changes enhance the adverse effects of oxidative stress, severely damaging retinal cells and promoting the development of macular edema, the primary symptom of diabetic retinopathy (DR), ultimately leading to vision loss in diabetic patients (Wang and Lo 2018; Wei et al. 2022).

Under normal physiological conditions, there is a balance between reactive oxygen/nitrogen species (ROS/RNS) and antioxidant agents to neutralize ROS/RNS. RPE cells contain several antioxidant enzymes, namely superoxide dismutase (SOD), catalase (CAT), and glutathione peroxidase (GPx) (He et al. 2017). Additionally, RPE cells possess natural nonenzymatic antioxidants, including glutathione (GSH), the most predominant antioxidant molecule in RPE cells (Sreekumar et al. 2021). However, during chronic hyperglycemia, the content of these endogenous antioxidants or the ability of RPE cells to counteract harmful molecules diminishes, leading to oxidative stress (Li et al. 2017). Therefore, it is crucial to determine whether maintaining a balance between free radicals and antioxidants can slow the pathological progression of this disease. Consequently, increasing antioxidant levels may be a viable strategy for protecting and therapeutic DR development.

Phytochemicals have gained attention as natural bioactive molecules that can potentially exert protective effects against diabetic retinopathy (Ola et al. 2017; Parveen et al. 2018). These compounds comprise nonnutrient secondary metabolites in fruits, vegetables, herbs, and spices. Previous studies have demonstrated that certain phytochemicals, such as phenolics, flavonoids, terpenoids, steroids, alkaloids, curcuminoids, and carotenoids, play various biological roles, mainly exhibiting antioxidant and anti‐apoptotic properties (Yu et al. 2021). Notably, anthocyanins, a group of natural bioactive compounds, have significant antioxidant and anti‐apoptotic potential and are abundantly present in vegetables, fruits, and herbs that display purple, pink, blue, and red colors (Khoo et al. 2017). Mulberry ( *Morus alba* L.), a genus of the *Morus* in the *Moraceae* family, is a fruit rich in anthocyanins (Ramappa et al. 2020). It is commonly grown and widely distributed across all regions of Thailand. Numerous pharmacological and biological studies of anthocyanins using animal and human models have been reported, highlighting their therapeutic properties. For instance, treatment with anthocyanins from bilberry has been shown to improve HO‐1 expression and significantly decrease the mRNA levels of IL‐6 and NF‐κB in the retina after light exposure in a rabbit model (Wang et al. 2019). Anthocyanin extract from blackcurrant significantly increases superoxide dismutase 1 (SOD1) levels in the retina following blue light‐induced damage in a mouse model (Shin et al. 2022). Anthocyanin from blueberry extract could increase CAT and GPx activities in RPE cells induced by cell damage using H_2_O_2_ (Huang et al. 2018). Previous research has demonstrated that mulberry fruit exhibits radical‐scavenging properties (Raman et al. 2016). Mulberry fruit extract has been shown to have radical‐scavenging activities against superoxide anion radicals and DPPH, including increased antioxidant systems, namely CAT and GSH (Wang et al. 2013). Therefore, mulberry extracts have the potential to prevent the DR development. This research aimed to evaluate mulberry extract's antioxidant effects and underlying mechanisms on high glucose‐induced oxidative injury in human retinal (differentiated ARPE‐19) cells.

## 2 Materials and Methods

### 2.1 Chemicals

All standards of anthocyanins (purity ≥ 99%), chemicals, and reagents were derived from Sigma‐Aldrich Co. (St. Louis, MO, USA). DMEM/F‐12, DMEM, and penicillin were purchased from Gibco (Life Technologies Corporation, Grand Island, NY 14072, USA). Fetal bovine serum (FBS) was obtained from Merck (Darmstadt, Germany). All antibodies were obtained from Cell Signaling (Danvers, USA).

### 2.2 Sample Preparation

Mulberry was procured from farms in Thailand's Chiang Mai, Rayong, and Nakhon Pathom provinces. The mulberries were thoroughly washed with water and then dried at room temperature. Subsequently, the dried mulberries were lyophilized, packed in aluminum foil under vacuum, and retained below –20°C until use.

### 2.3 Sample Extraction

Equal amounts of dried samples from each province were pooled and homogenized. One gram of the freeze‐dried sample was soaked and extracted with 15 mL of 90% ethanol, centrifuged at 4600 *g* for 15 min at RT, and evaporated till dry using a rotary evaporator under vacuum at 35°C–40°C. The dried extract film was retained below −20°C until use. This mulberry extract is referred to as ME.

### 2.4 Cell Culture

Human retina (ARPE‐19) cells from ATCC (Rockville, MD, USA) were maintained in a DMEM/F‐12 containing heat‐inactivated FBS and penicillin–streptomycin at the ratio 10% and 1% (v/v), respectively. All experiments used cells in passage numbers between 11 and 15 with over 90% confluency. Differentiated ARPE‐19 cells were used to represent human RPE cells. The differentiation of ARPE‐19 cells into a more native and physiologically relevant state was achieved by maintaining confluent cells in DMEM medium containing high glucose (4.5 g/L), supplemented with 1% heat‐inactivated FBS, 1% 100 mM sodium pyruvate, and 1% 200 mM L‐glutamine for 2 months (Samuel et al. 2017). The culture medium was changed three times per week.

### 2.5 MTT Assay

The viability of the cell was determined using the MTT assay. Once the cells reached their designated experimental time points, the media was discarded, and the cells were washed once with PBS. The cells were then treated with an MTT solution at a concentration of 0.5 mg/mL in PBS at 37°C for 3 h. DMSO was added to dissolve the formazan crystals. The yellow MTT is converted into purple formazan, which correlates with the number of viable cells. The viability of the cells was assessed by the determination of OD at 540 nm using a microplate reader.

### 2.6 Evaluation of Cytotoxicity of ME and Glucose

Differentiated ARPE‐19 cells were cultured at 0.1 × 10^6^ cells per well in a 48‐well plate for 24 h; cells were washed once with DMEM medium. For the cytotoxicity assessment of mulberry extract (ME), the cells were incubated with ME at 100, 200, 500, 1000, and 2000 μg/mL for 72 h, with 0.5% DMSO as a vehicle control. For the cytotoxicity assessment of glucose, the cells were treated with glucose at 10, 20, 25, 50, and 100 mM for 24 and 48 h, with DMEM medium serving as the control group. The viability of the cells was assessed using the MTT assay.

### 2.7 Evaluation of the Effect of ME on the Viability of Differentiated ARPE‐19 Cells Exposed to Glucose‐Induced Oxidative Injury

Differentiated ARPE‐19 cells were seeded at 1.0 × 10^6^ cells and 0.1 × 10^6^ cells per well in the 6‐well and 48‐well plates, respectively, for 24 h. The cells were washed once with DMEM and treated with ME at concentrations determined to be nontoxic from Section 2.6 in DMEM for 24 h. A 0.5% DMSO in DMEM was used as a control. After treatment with ME, the cells were washed once with DMEM and then incubated with glucose in DMEM at appropriate concentrations and durations (as specified in Section 2.6). Cell viability was measured using the MTT assay in the 48‐well plates. The assay kit and western blot analysis determined critical enzyme activities, protein expression of antioxidants, and apoptosis markers from cells cultured in six‐well plates.

### 2.8 Evaluation of the Effect of ME on the ROS Production in Differentiated ARPE‐19 Cells Exposed to Glucose‐Induced Oxidative Injury

Differentiated ARPE‐19 cells were cultured into black 96‐well plates with a clear bottom (Corning, NY, USA) at a density of 3.5 × 10^4^ cells per well. The cells were washed with DMEM and pretreated with ME (at concentrations determined to be nontoxic from Section 2.6) in DMEM for 24 h. Following incubation with ME, the cells were washed and then treated with glucose in DMEM at appropriate concentrations and durations (as specified in Section 2.6). A 0.5% DMSO was used as a control. After incubation, the cells were washed with DMEM and incubated with 0.01 mM DCFH‐DA in DMEM media at 37°C for 20 min. ROS production was determined using a fluorescence plate reader with an excitation of 485 nm and an emission of 530 nm.

### 2.9 Evaluation of the Effect of ME on Activities of SOD, CAT, and GPx, and Levels of GSH in Differentiated ARPE‐19 Cells Exposed to Glucose‐Induced Oxidative Injury

Following pretreatment with ME and induction with glucose, as described in Section 2.7, cells were soaked and scraped with PBS containing 1% (v/v) Triton X‐100. The cell suspension was then sonicated in an ultrasonic sonicator at 4°C for 10 min. The cell lysates were centrifuged at 12,500 *g*, 4°C for 10 min. The supernatants were collected to determine SOD, GPx, and CAT activities, as well as GSH levels, using assay kits (Cayman Chemical, Michigan, USA).

### 2.10 Evaluation of the Effect of ME on Caspase‐9 and ‐3 Activities in Differentiated ARPE‐19 Cells Exposed to Glucose‐Induced Oxidative Injury

Following pretreatment with ME and induction with glucose from Section 2.7, the cells were combined in a hypotonic lysis buffer to derive the supernatant. A specific substrate of caspase‐9 and caspase‐3 was added to the supernatant at a concentration of 100 μmol/L. The solution mixture was incubated at 37°C for 60 min before measuring OD at 450 nm.

### 2.11 Evaluation of the Effect of ME on Apoptosis Protein Expression in Differentiated ARPE‐19 Cells Exposed to Glucose‐Induced Oxidative Injury

Following pretreatment and induction with ME and glucose, as described in Section 2.7, protein lysate from cells was provided using cell lysis buffer and centrifuged at 12,500 *g*, 4°C for 10 min. The supernatants were kept, and protein concentration was determined using the BCA assay. 10% SDS‐PAGE separated equal amounts (40 μg) of protein samples. The resolved proteins were transferred to a nitrocellulose membrane and then blocked with 5% skim milk. The membrane was incubated overnight with 0.1% (v/v) anti‐Bax, anti‐cytochrome *c*, or anti‐Bcl‐2 antibodies. After that, the membrane was incubated with the secondary antibody (0.05% v/v) for 120 min. The membrane was exposed to X‐ray film, and the target band densities were measured using the ImageJ software. Our results represented the relative band intensity ratio of the target bands to beta‐actin.

### 2.12 Evaluation of the Effect of ME on Nrf2 Level in Differentiated ARPE‐19 Cells Exposed to Glucose‐Induced Oxidative Injury

ARPE‐19 was cultured in six‐well plates and incubated for 24 h. The cells were treated with ME (at concentrations determined to be nontoxic from Section 2.6) in DMEM for 24 h. A 0.5% DMSO in DMEM was used as a control. The cells were rinsed with cold PBS, and cytosolic and nuclear proteins were isolated using the Cell Fractionation Kit (Cell Signaling, Danvers, USA). Protein concentrations were measured with BCA (Thermo Scientific, Rockford, the USA). The expression levels of cytosolic and nuclear proteins were normalized to the intensity of beta‐actin and Lamin B, respectively.

## 3 Results

### 3.1 Anthocyanin in ME

**FIGURE 1:**
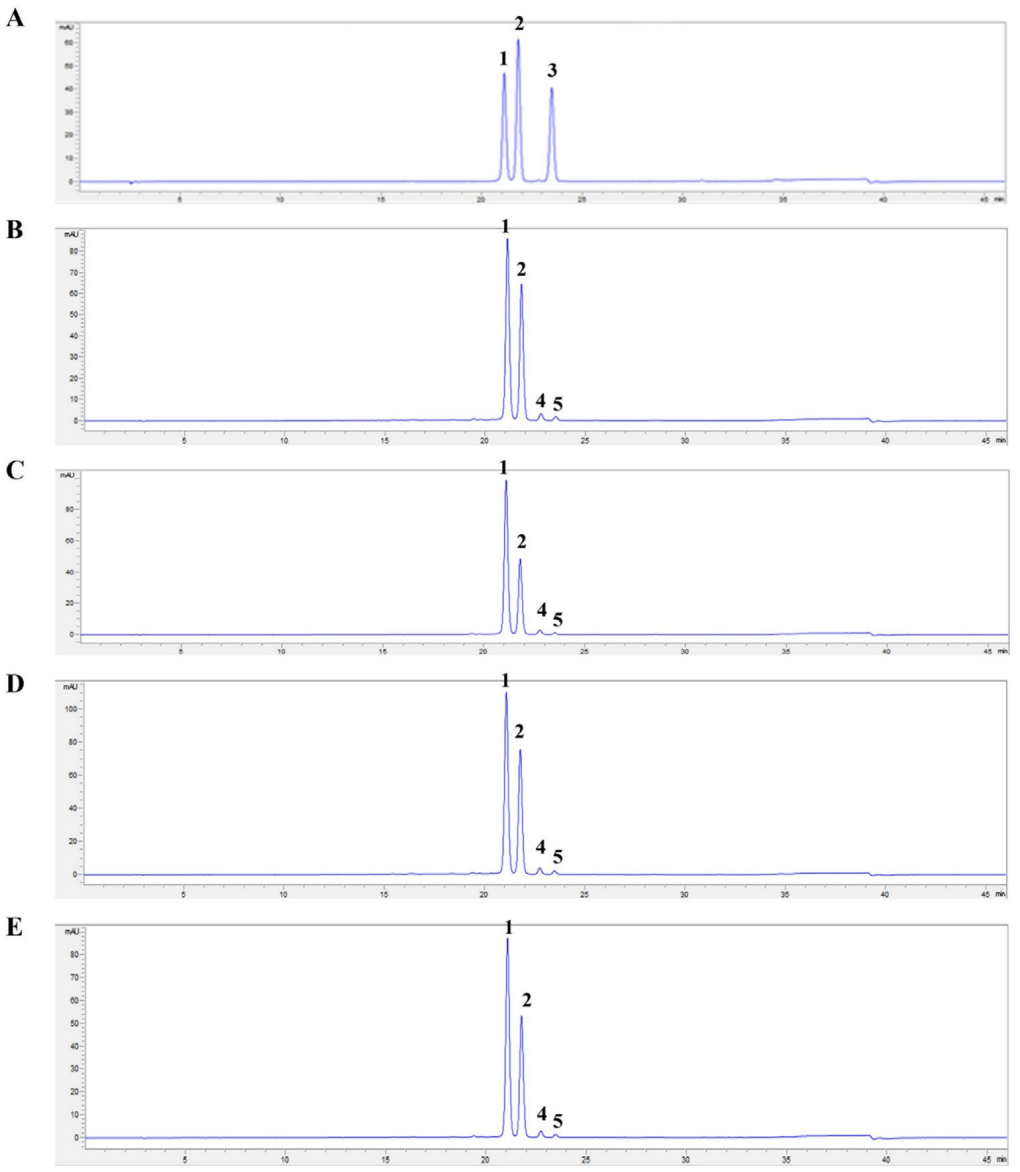
Chromatograms of anthocyanin from (A) mixed standard, (B) mulberry fruit from Nakhon Pathom, (C) mulberry fruit from Rayong, (D) mulberry fruit from Chiang Mai, and (E) mixed mulberry fruit from three provinces. 1 = cyanidin‐3‐glucosides (C3G), 2 = cyanidin‐3‐rutinosides (C3R), 3 = peonidin‐3‐glucosides (P3G), and 4 and 5 = unknown anthocyanins.

Our studies revealed that the treatment with ME provided the most significant protective effects against glucose‐induced oxidative injury resulting in cell death. Consequently, the phytochemical constituents of ME were analyzed using HPLC to identify the compounds responsible for enhancing antioxidant defense and preventing apoptosis in ARPE‐19 cells. Figure 1 shows the chromatogram of anthocyanin in mulberry fruits collected from three different provinces, including Nakhon Pathom, Rayong, and Chiang Mai, and the mixture of them. The two prominent peaks, 1 and 2, corresponded to C3G and C3R. The amount of C3G and C3R in mulberry fruits varied across provinces, with Nakhon Pathom containing 7.85 and 9.93 mg/g of dry weight, Rayong showing 7.35 and 9.57 mg/g of dry weight, and Chiang Mai showing 9.78 and 10.52 mg/g of dry weight, respectively. The mixed mulberry fruits from the three provinces contained 8.13 and 9.86 mg/g of dry weight, showing no significant differences. Mulberry fruits from three provinces contain two minor anthocyanins, which led to the mixed mulberry fruits also having these.

### 3.2 Long‐Term Differentiation of ARPE‐19 Cells Results in a Phenotype That Exhibits More Native RPE Characteristics Compared to Undifferentiated ARPE‐19 Cells

**FIGURE 2:**
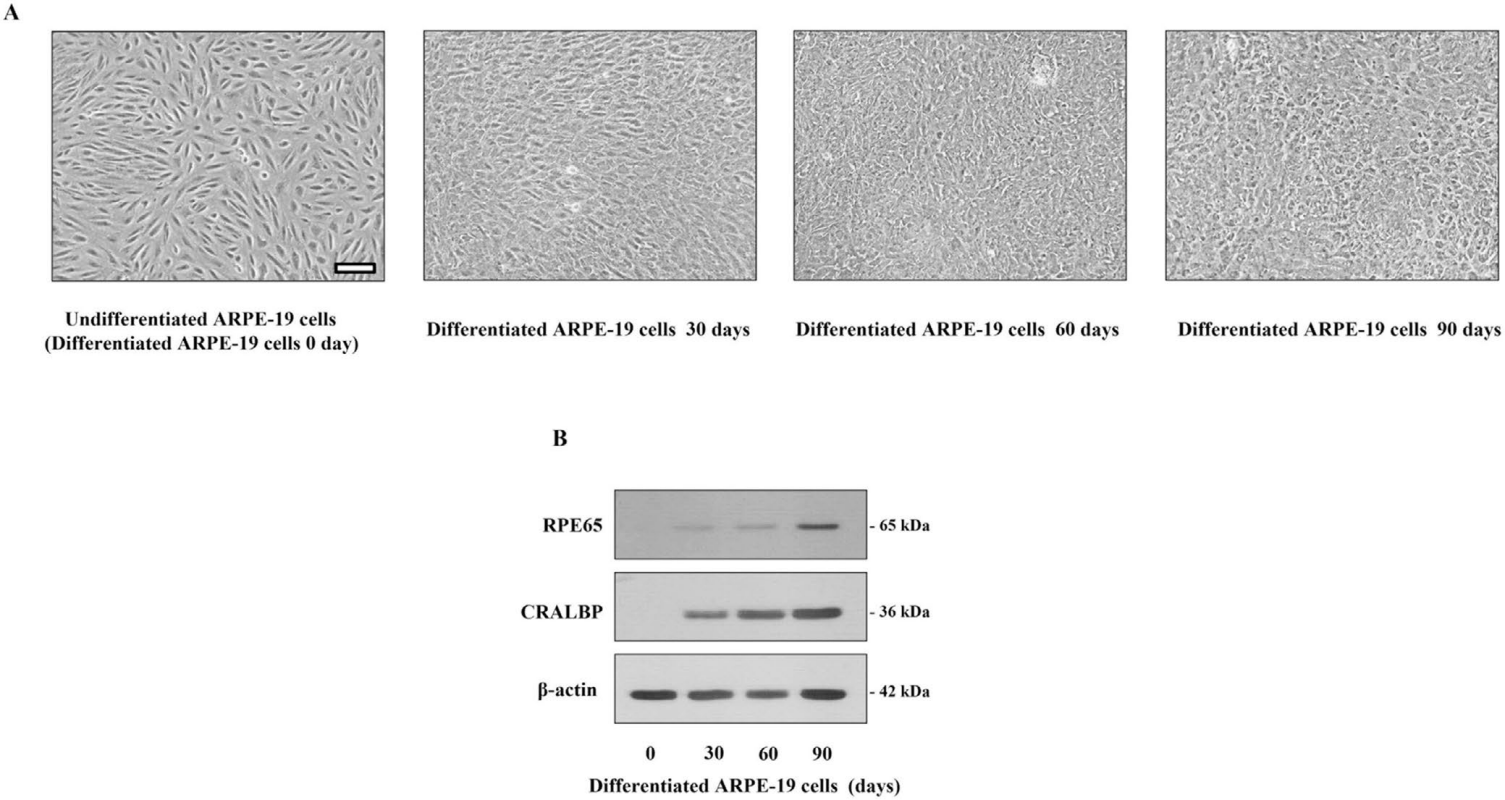
(A) Phase–contrast microscopy images show the morphology of undifferentiated ARPE‐19 cells and those differentiated for 30, 60, and 90 days. The scale bar represents 100 μm. (B) Protein expression levels of RPE‐specific markers, RPE65 and CRALBP, were analyzed by immunoblotting in both undifferentiated and differentiated ARPE‐19 cells. β‐Actin was used as a loading control for normalization.

Previous studies showed that ARPE‐19 cells cultured over 3 months developed a phenotype characteristic of native retinal pigment epithelium (RPE) and expressed proteins, mRNAs, and miRNAs associated with RPE identity. Comparative analysis of the ARPE‐19 RNA‐Seq dataset with primary human fetal RPE, embryonic stem cell‐derived RPE, and native RPE revealed a substantial overall similarity in gene expression patterns across all models and native tissue (Ahmado et al. 2011; Dunn et al. 1996; Samuel et al. 2017). Based on the results, ARPE‐19 cells that were differentiated for 3 months displayed a more pronounced cobblestone morphology and were more tightly packed than undifferentiated cells, which exhibited an elongated, fibroblast‐like appearance (Figure 2A). Recent studies have shown that media conditions and extended culture periods enable ARPE‐19 cells to achieve a more native‐like state. In the present study, ARPE‐19 cells were cultured in specialized differentiation DMEM media for 30, 60, and 90 days compared to those grown in standard DMEM/F12 media (0 days). Cells differentiated for 90 days exhibited the highest expression of the retinal pigment epithelium‐specific 65 kDa protein (RPE65) and cellular retinaldehyde‐binding protein (CRALBP), as determined by immunoblotting (Figure 2B). This indicates that differentiation enhances the expression of these key RPE markers, reflecting a more differentiated and specialized state that is more physiologically relevant to native RPE cells. Thus, this study selected a differentiation period of 90 days (or 3 months) for all subsequent tests.

### 3.3 Effect of ME on Cell Viability of Differentiated ARPE‐19 Cells

**FIGURE 3:**
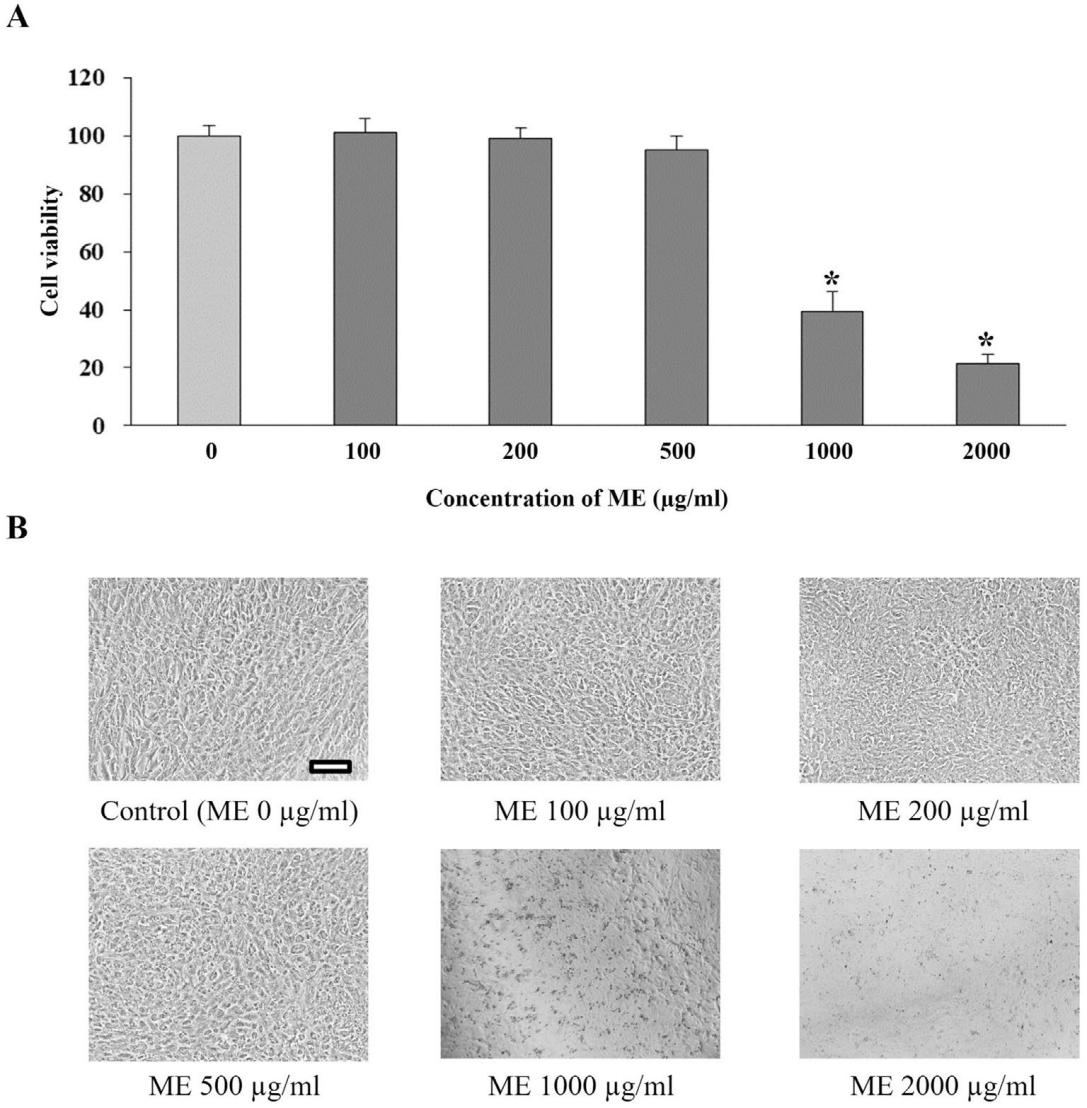
Effects of ME on the viability of differentiated ARPE‐19 cells. (A) Differentiated ARPE‐19 cells were incubated with ME from 100 to 2000 μg/mL in a DMEM medium for 72 h, after which the viability of cells was evaluated by MTT assay. 0. 50.5% DMSO in DMEM medium was used as the control group. Graphs represent average cell viability (mean ± SD; *n* = 3). Symbol * indicates significantly different from the control group (*p* < 0.05). (B) Morphology by phase–contrast microscopy of differentiated ARPE‐19 cells under all experimental conditions. The scale bar represents 100 μm, 10× magnification.

The effect of ME on the viability of cells was determined. Differentiated ARPE‐19 was cultured with several concentrations of ME (100, 200, 500, 1000, and 2000 μg/mL) for 72 h. We found that the viability of cells treated with ME at concentrations greater than 500 μg/mL for 72 h was significantly lower than that of the control group (*p* < 0.05) (Figure 3A). These results indicated that the concentration of ME greater than 500 μg/mL was toxic to differentiated ARPE‐19 cells. Inverted microscope observation (Figure 3B) confirmed the cytotoxic effects of ME. Thus, the highest ME concentration at 500 μg/mL was selected for future experiments to explore its preventive properties on differentiated ARPE‐19 cells.

### 3.4 Effect of High Glucose on Cell Viability of Differentiated ARPE‐19 Cells

**FIGURE 4:**
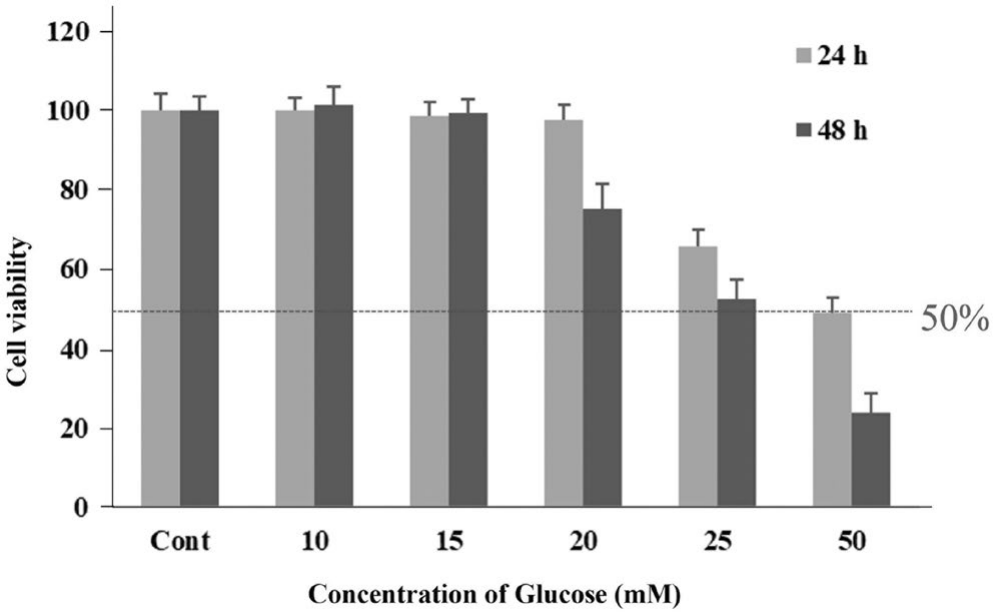
Effects of glucose on the viability of differentiated ARPE‐19 cells. The cells were incubated with glucose from 10 to 50 mM in DMEM medium for 24 and 48 h, after which the viability of the cells was evaluated by MTT assay. 0.5% DMSO in DMEM medium was used as the control group. Graphs represent average cell viability (mean ± SD; *n* = 3). Symbol * indicates significantly different from the control group (*p* < 0.05).

The cytotoxic dose of glucose on the viability of ARPE‐19 cells was determined. Differentiated ARPE‐19 was treated with glucose at 10, 15, 20, 25, and 50 mM for 24 and 48 h. The experimental findings indicated that the cell survival rate for those treated with over 25 mM glucose for 24 and 48 h was significantly lower than that of the control group (*p* < 0.05). Exposing cells to 25 mM glucose for 48 h resulted in a 50% reduction in cell viability (Figure 4). Therefore, the suitable dose and time of glucose for oxidative injury induction in differentiated ARPE‐19 cells were 25 mM and 48 h, respectively.

### 3.5 Effect of ME on the Viability of Differentiated ARPE‐19 Cells Induced by Oxidative Injury With High Glucose

**FIGURE 5:**
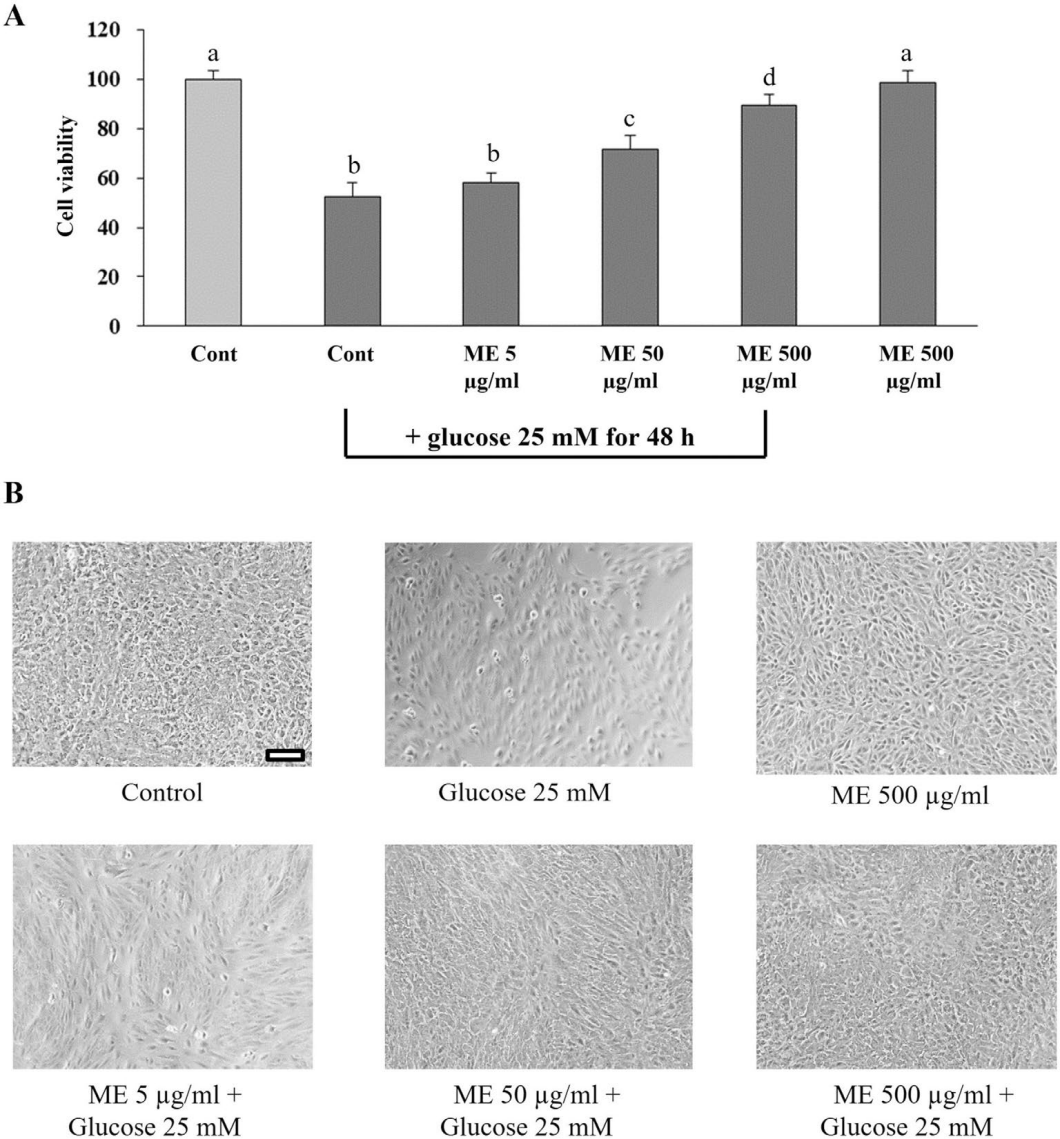
Effects of ME on the viability of differentiated ARPE‐19 cells induced oxidative injury with high glucose. (A) Cells were incubated with ME at 5, 50, and 500 μg/mL in DMEM medium for 24 h, followed by glucose induction at 25 mM for 48 h. The viability of cells was evaluated using an MTT assay. 0. 50.5% DMSO in DMEM medium was used as the control group. Graphs represent average cell viability (mean ± SD; *n* = 3). The letters (a–d) above the error bars indicate significant differences among groups at *p* < 0.05. (B) Morphology by phase–contrast microscopy of differentiated ARPE‐19 cells under all experimental conditions. The scale bar represents 100 μm, 10× magnification.

We also evaluated the preventive effects of ME against oxidative injury in differentiated ARPE‐19 cells exposed to 25 mM glucose for 48 h. We found that glucose showed a 50% reduction in cell viability compared to the control group (Figure 5A). Preincubating cells with 50 and 500 μg/mL of ME for 24 h significantly (*p* < 0.05) improved the viability of differentiated ARPE‐19 cells depending on the amount of ME compared to the glucose group (cells incubated with glucose alone), as shown in Figure 5A. However, pretreating cells with 5 μg/mL of ME for 24 h showed no difference in the viability of differentiated ARPE‐19 cells compared to the glucose group (cells treated with glucose alone) (Figure 5A). Inverted microscopic analysis (Figure 5B) supported the preventive effect of ME against glucose‐induced oxidative injury in differentiated ARPE‐19 cells. These findings suggest that ME at 50 and 500 μg/mL could attenuate differentiated ARPE‐19 cells from glucose‐induced oxidative injury, depending on the amount of ME.

### 3.6 Effect of ME on ROS Generation in Differentiated ARPE‐19 Cells Induced Oxidative Injury With High Glucose

**FIGURE 6:**
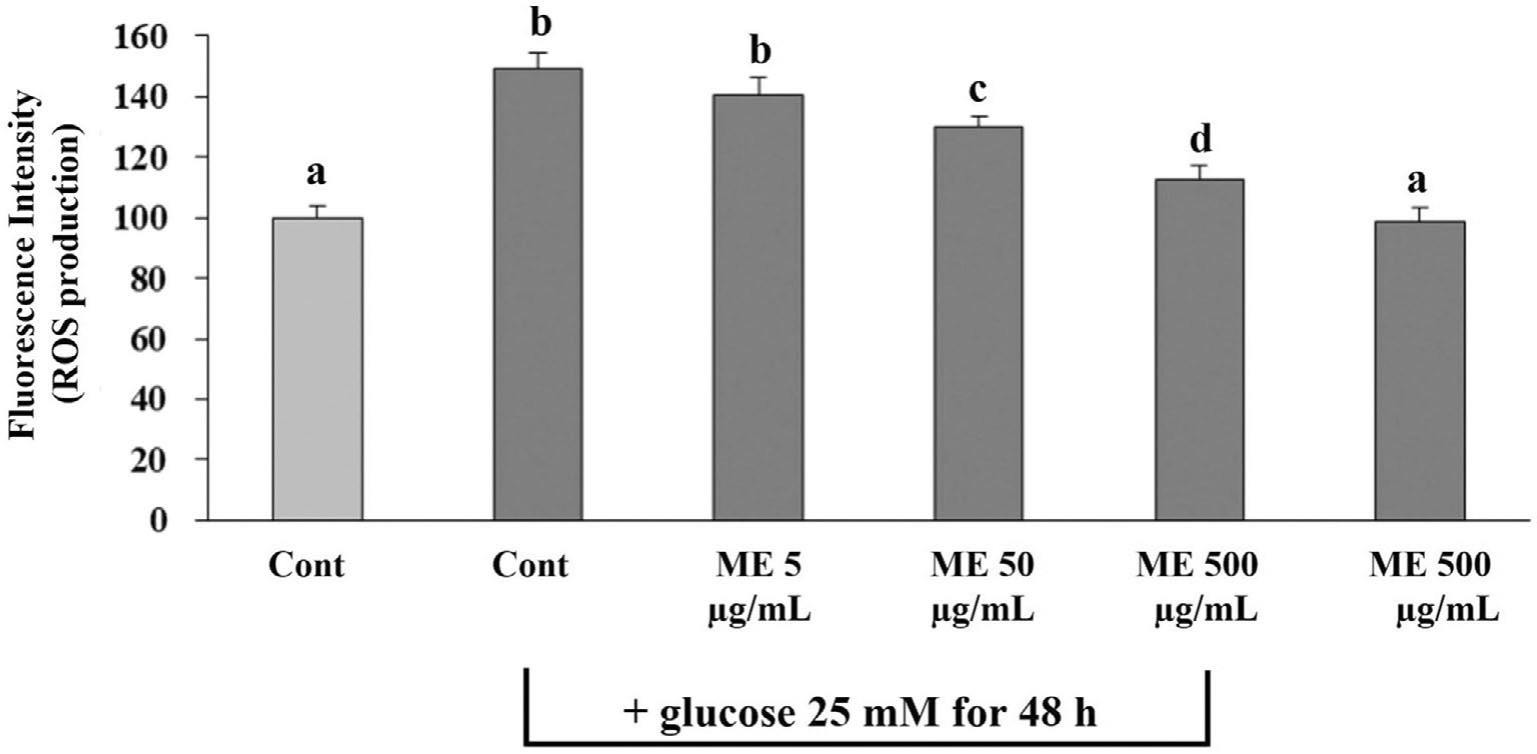
Effects of ME on ROS production in differentiated ARPE‐19 cells induced oxidative injury with high glucose. Differentiated ARPE‐19 cells were incubated with ME at 5, 50, and 500 μg/mL in DMEM for 24 h, followed by glucose induction at 25 mM for 48 h. ROS production was evaluated by DCFH‐DA assay. 0.5% DMSO in DMEM medium was used as the control group. Graphs represent average cell viability (mean ± SD; *n* = 3). The letters (a–d) above the error bars indicate significant differences among groups at *p* < 0.05.

We examined the protective effects of ME on ROS generation in differentiated ARPE‐19 cells exposed to glucose. Cells exposed to glucose showed significantly (*p* < 0.05) escalated ROS generation in comparison with the control group (Figure 6). ME at 50 and 500 μg/mL was a significant (*p* < 0.05) decrease in ROS generation when compared with the glucose group depending on the amount of ME (Figure 6). However, the incubation of cells with 5 μg/mL of ME for 24 h showed no difference in the ROS level of differentiated ARPE‐19 cells compared to the glucose group (cells were treated with glucose alone) (Figure 6). These results suggest that ME at 50 and 500 μg/mL could prevent and attenuate differentiated ARPE‐19‐induced cell injury with glucose by reducing ROS production depending on the amount of ME.

### 3.7 Effect of ME on Activities of Caspase‐3 and ‐9 in Differentiated ARPE‐19 Cells Induced Oxidative Injury With Glucose

**FIGURE 7:**
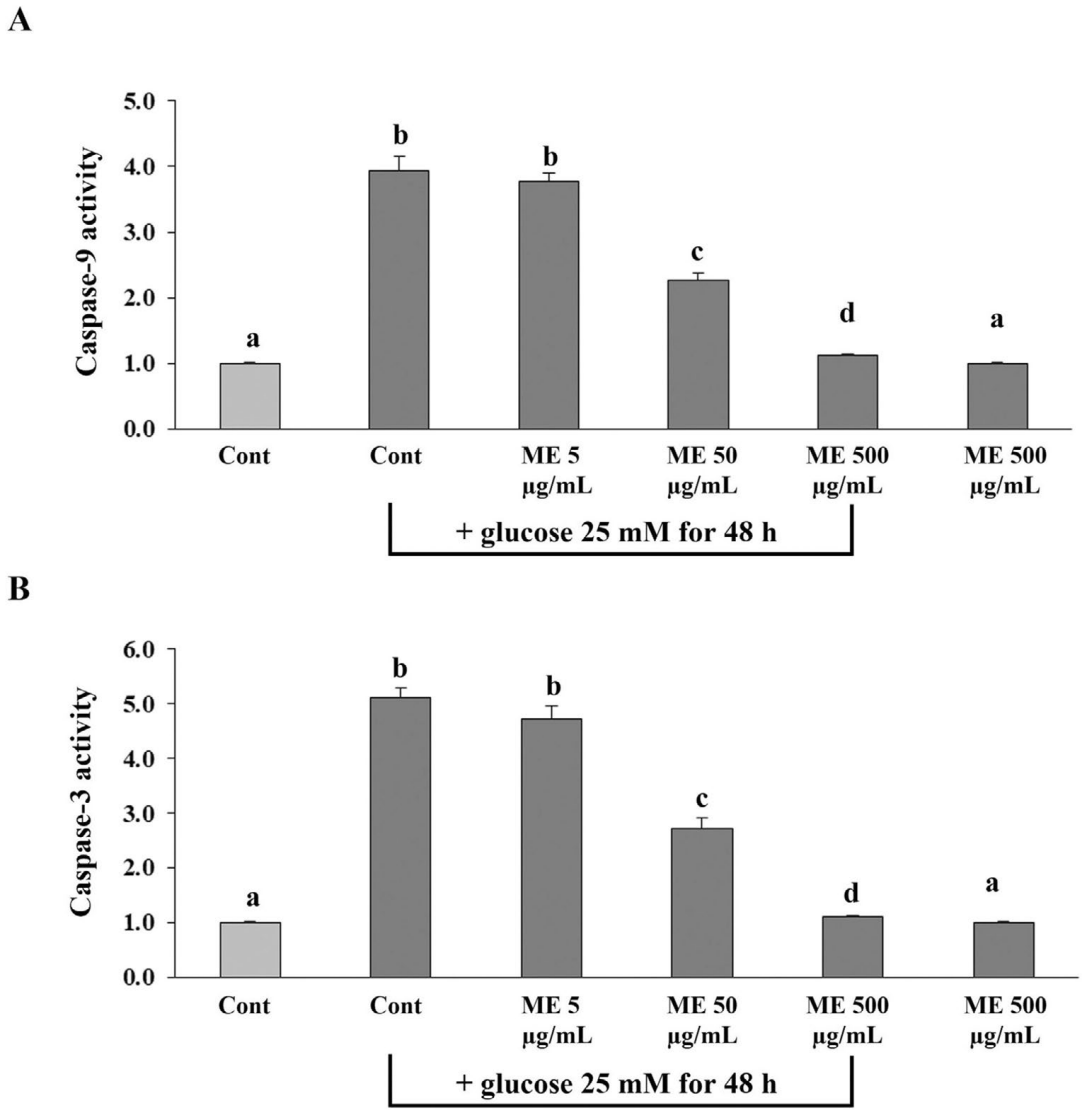
Effects of ME on caspase–9 and –3 activities in differentiated ARPE–19 cells induced oxidative injury with glucose. Cells were pretreated with ME at concentrations of 5, 50, and 500 μg/mL for 24 h. After incubation, cells were induced oxidative injury with glucose at the concentration of 25 mM for 48 h. The treated cells were homogenized in a hypotonic buffer to obtain the part of the supernatant. The supernatant was determined (A) caspase–9 and (B) caspase–3 activities. 0.5% DMSO in DMEM medium was used as the control group. Graphs represent average caspase activities (mean ± SD; *n* = 3). The letters (a–d) above the error bars indicate significant differences among groups at *p* < 0.05.

To assess the anti‐apoptotic properties of ME in oxidative injury induced in differentiated ARPE‐19 cells, the activities of caspases (9 and 3) were evaluated. Cells that were incubated with only glucose showed significantly (*p* < 0.05) increased activity of caspase‐9 and caspase‐3 compared to the control group (Figure 7A,B). However, the treatment with ME at 50 and 500 μg/mL significantly decreased the activities of caspase‐9 and caspase‐3 depending on the amount of ME (Figure 7A,B). The results indicate that ME could mitigate glucose‐induced apoptosis in differentiated ARPE‐19 cells by regulating the activities of key caspases (9 and 3) in the apoptotic pathway.

### 3.8 Effect of ME on Apoptosis Markers’ Expression in Differentiated ARPE‐19 Cells Induced by Oxidative Injury With Glucose

**FIGURE 8:**
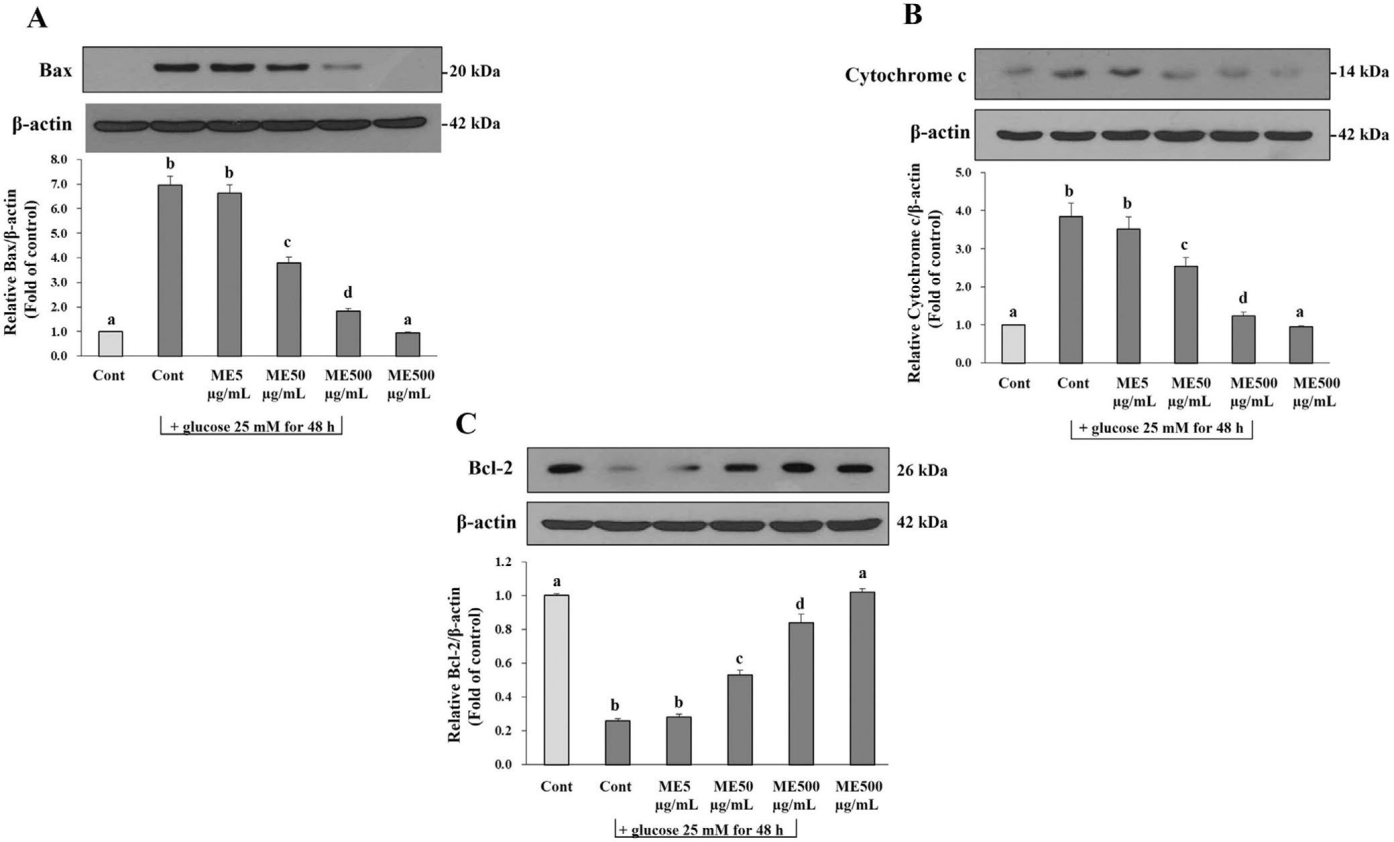
Effects of ME on apoptosis markers expression in differentiated ARPE‐19 cells induced oxidative injury with glucose. Cells were pretreated with ME at concentrations of 5, 50, and 500 μg/mL for 24 h. After incubation, cells were induced oxidative injury with glucose at the concentration of 25 mM for 48 h. The treated cells were homogenized in a hypotonic buffer to obtain the part of the supernatant. The supernatant was determined (A) Bax, (B) Cytochrome *c*, and (C) Bcl‐2 protein expression. Results are reported as mean ± SD of at least three independent experiments. Different superscript letters indicate significant differences between values in the column (*p* < 0.05).

We also proved the effect of ME on key apoptosis markers of the apoptosis pathway. Immunoblotting identified Bax, Bcl‐2, and cytochrome *c* expression levels (Figure 8A–C). The analysis revealed that glucose induction significantly (*p* < 0.05) escalated the levels of Bax and cytochrome *c* expression while significantly (*p* < 0.05) decreasing the levels of Bcl‐2 expression compared with the control group (Figure 8A–C). However, the incubation with ME at 50 and 500 μg/mL significantly (*p* < 0.05) reduced the expression of Bax and cytochrome *c* while significantly (*p* < 0.05) increasing the expression of Bcl‐2 depending on the amount of ME (Figure 8A–C). The results indicated that ME could improve glucose‐induced apoptosis in differentiated ARPE‐19 cells by improving the crucial apoptotic protein markers in the apoptosis signaling pathway.

### 3.9 Effect of ME on SOD, CAT, and GPx Activities, and GSH Levels in Differentiated ARPE‐19 Cells Induced Oxidative Injury With Glucose

**FIGURE 9:**
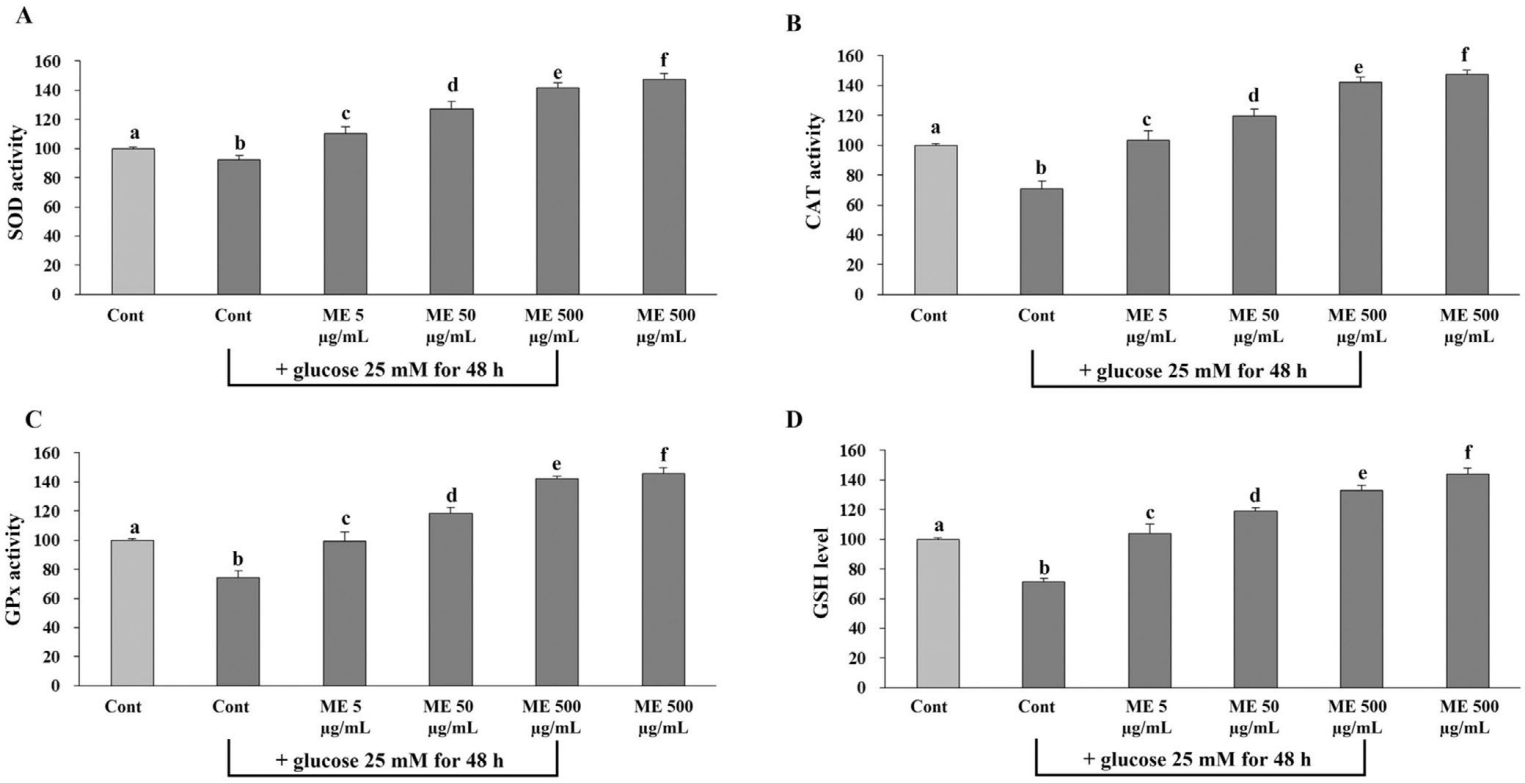
Effects of ME on SOD, CAT, and GPx activities, and GSH levels in differentiated ARPE‐19 cells induced oxidative injury with glucose. Cells were pretreated with ME at concentrations of 5, 50, and 500 μg/mL for 24 h. After incubation, cells were induced oxidative injury with glucose at the concentration of 25 mM for 48 h. The treated cells were homogenized in a hypotonic buffer to obtain the part of the supernatant. The supernatant was determined (A) SOD, (B) CAT, (C) GPx activities, and (D) GSH level. Results are reported as mean ± SD of at least three independent experiments. Different superscript letters indicate significant differences between values in the column (*p* < 0.05).

Enzymatic antioxidants, including SOD, CAT, and GPx, along with nonenzymatic antioxidants like GSH, protect against oxidative injury by neutralizing excessive levels of ROS in cells. Therefore, we investigated whether ME modulates the capacity of intracellular antioxidants (SOD, CAT, GPx, and GSH) to protect differentiated ARPE‐19 cells against oxidative injury. The results showed that glucose treatment significantly (*p* < 0.05) reduced the ability of intracellular antioxidants compared to the untreated control group (Figure 9A–D). However, preincubation with ME significantly enhanced the capacity of intracellular antioxidants in differentiated ARPE‐19 cells exposed to glucose, which causes oxidative injury (Figure 9A–D). Interestingly, ME treatment alone significantly enhanced the efficacy of intracellular antioxidants of differentiated ARPE‐19 cells compared to the control (Figure 9A–D). The findings suggest that ME may enhance the intracellular antioxidant response of RPE cells, thereby improving their ability to withstand potential oxidative stressors. In addition, it was highlighted that ME boosts the capacities of the intracellular antioxidant system of differentiated ARPE‐19.

### 3.10 Effect of ME on Nrf2 Expression in Differentiated ARPE‐19 Cells Induced Oxidative Injury With Glucose

**FIGURE 10:**
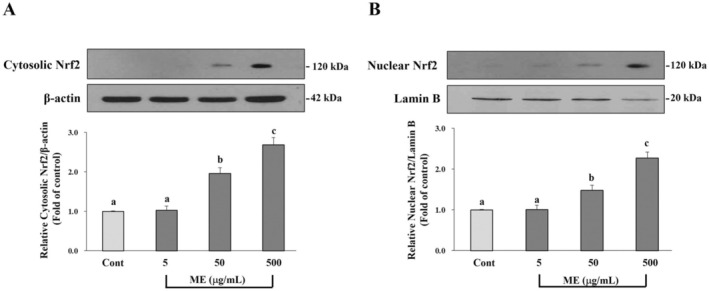
Effects of ME on Nrf2 expression in differentiated ARPE‐19 cells exposed to glucose‐induced oxidative injury. Differentiated ARPE‐19 cells were pretreated with ME at concentrations of 5, 50, and 500 μg/mL for 24 h. Western blot analysis showed the expression of the active form of Nrf2 in (A) cytosolic and (B) nuclear fractions. Results are reported as mean ± SD of at least three independent experiments. Different superscript letters indicate significant differences between values in the column (*p* < 0.05).

We also explored the transcription factor Nrf2, a key signaling factor that controls the working of the intracellular antioxidant systems. Differentiated ARPE‐19 was maintained with ME for 24 h, after which the level of the active form of Nrf2 protein was examined. The analysis from the western blot demonstrated that the treatment of ME significantly enhanced the expression of the active form of Nrf2 in both cytosolic and nuclear fractions, depending on the amount of ME compared to the control group (*p* < 0.05) (Figure 10A,B). Our results suggested that ME activated Nrf2 and promoted its translocation to the nucleus. This nuclear translocation of Nrf2 subsequently led to the upregulation of both enzymatic and nonenzymatic antioxidants, thereby strengthening the cell's antioxidant defense system. The activation of Nrf2 likely contributed to controlling key protective genes involved in the oxidative injury response, enhancing cellular resilience against oxidative injury.

## 4 Discussion

DR is a diabetes complication that is the primary cause of visual dysfunction and irreversible vision loss globally. The progression of DR is linked to a persistent metabolic disorder resulting from hyperglycemia. Numerous studies showed that oxidative stress from exposure to high glucose is a significant cause of RPE cell dysfunction and death caused by the development of DR (Wei et al. 2022). The RPE is a uniquely polarized epithelial cell via a substantial level of specialization that communicates with photoreceptors in the macula. Moreover, RPE cells are crucial in supporting regular sight because of their outstanding and varied functions. The developmental steps guide the definition and differentiation of the RPE for improved knowledge of the mechanism underlying the RPE's ability. Thus, preventing RPE cells from high glucose‐stimulated oxidative injury may be an effective protective and therapeutic strategy against retinal degenerative disorders, including DR development. Several studies used ARPE‐19 cells to represent RPE cells for models of eye diseases because the differentiation of ARPE‐19 cells can express RPE‐specific markers, including RPE65 and CRALBP, that achieve phenotypic characteristics closer to those of native RPE cells, especially when studying retinal pathophysiology and treatment effects (Dunn et al. 1996; Samuel et al. 2017). Our findings show that ARPE‐19 cells differentiated for 90 days exhibited the highest expression levels of RPE65 and CRALBP, as confirmed by immunoblotting (Figure 2B). This suggests that prolonged differentiation enhances the expression of these key RPE markers, indicating a more specialized and physiologically relevant state resembling native RPE cells. Consequently, a differentiation period of 90 days was selected for all subsequent experiments in this study.

Our understanding is that this research is the original to demonstrate the protective and attenuative effects underlying mechanisms of ME against oxidative injury from glucose induction in human RPE cells, serving as a model for DR. We found that ME could shield differentiated ARPE‐19 cells from oxidative injury induced by high glucose conditions by reducing ROS levels. Mechanistically, this protective effect was associated with increased Bax and cytochrome *c* protein levels and decreased Bcl‐2 protein levels. Our findings demonstrated that the cell death observed in RPE cells following glucose induction occurred via the apoptotic pathway. The treatment with ME at 50 and 500 μg/mL for 24 h prevented these apoptotic changes, enhancing cell viability against oxidative injury in this RPE cell model. The biological activity of ME is likely attributed to the presence of anthocyanins, particularly C3G and C3R, as indicated by HPLC analysis. Numerous studies have shown that various anthocyanins, including C3G and C3R, could promote the translocation of Nrf2 into the nucleus (Sukprasansap et al. 2020; You et al. 2017). This activation triggers the gene expression of several antioxidant enzymes and phase I and II detoxification enzymes, which may help safeguard against the development of DR caused by oxidative injury (Jomova et al. 2023). Many studies have found that general dietary anthocyanin consumption can reduce degenerative disease growth through its antioxidant properties (Bendokas et al. 2020; Escalante‐Aburto et al. 2023). Additionally, our experiment demonstrated that exposure of differentiated ARPE‐19 cells to high glucose levels resulted in oxidative injury by significantly decreasing both the activities and the levels of intracellular antioxidants (SOD, CAT, GPx, and GSH). In contrast, cells were given ME at 50 and 500 μg/mL for 24 h following glucose induction; the results showed a significant increase in both the activities and the levels of intracellular antioxidants. Moreover, in normal conditions (in the absence of oxidative injury), ME treatment significantly enhanced the activities and the levels of intracellular antioxidants compared to the control cells. These findings suggest that ME treatment may shield RPE cells against oxidative injury by boosting the capacity of the intracellular antioxidant system of human retina cells.

Numerous studies have demonstrated that the natural antioxidants could attenuate oxidatively stressed and injured RPE cells by motivating the crucial transcription factor Nrf2, which links to the translocation of Nrf2 from the cytoplasm into the nucleus, leading to the switch‐on of various intracellular antioxidant expressions. Many research studies have shown that anthocyanin, including C3G and C3R, protects against oxidative injury and damage by activating the Nrf2 transcription factor (Herrera‐Bravo et al. 2022; Yu et al. 2021). Additionally, our study found that ME enhanced the levels of the active form of Nrf2 in both cytosolic and nuclear fractions. This suggests that ME not only activates Nrf2 but also promotes its translocation to the nucleus. The translocation of Nrf2 into the nucleus subsequently leads to the turn of both enzymatic and nonenzymatic antioxidant expression, thereby strengthening the cell's antioxidant defense system. Therefore, the natural bioactive compounds in ME, particularly the anthocyanins C3G and C3R, may prevent and alleviate damaged human retinal cells from glucose‐induced oxidative injury, which is a key factor in the progression of DR by modulating the essential intracellular antioxidant by enhancing the intracellular antioxidants of human retina cells through the activation of the Nrf2 transcription factor.

## 5 Conclusions

**FIGURE 11:**
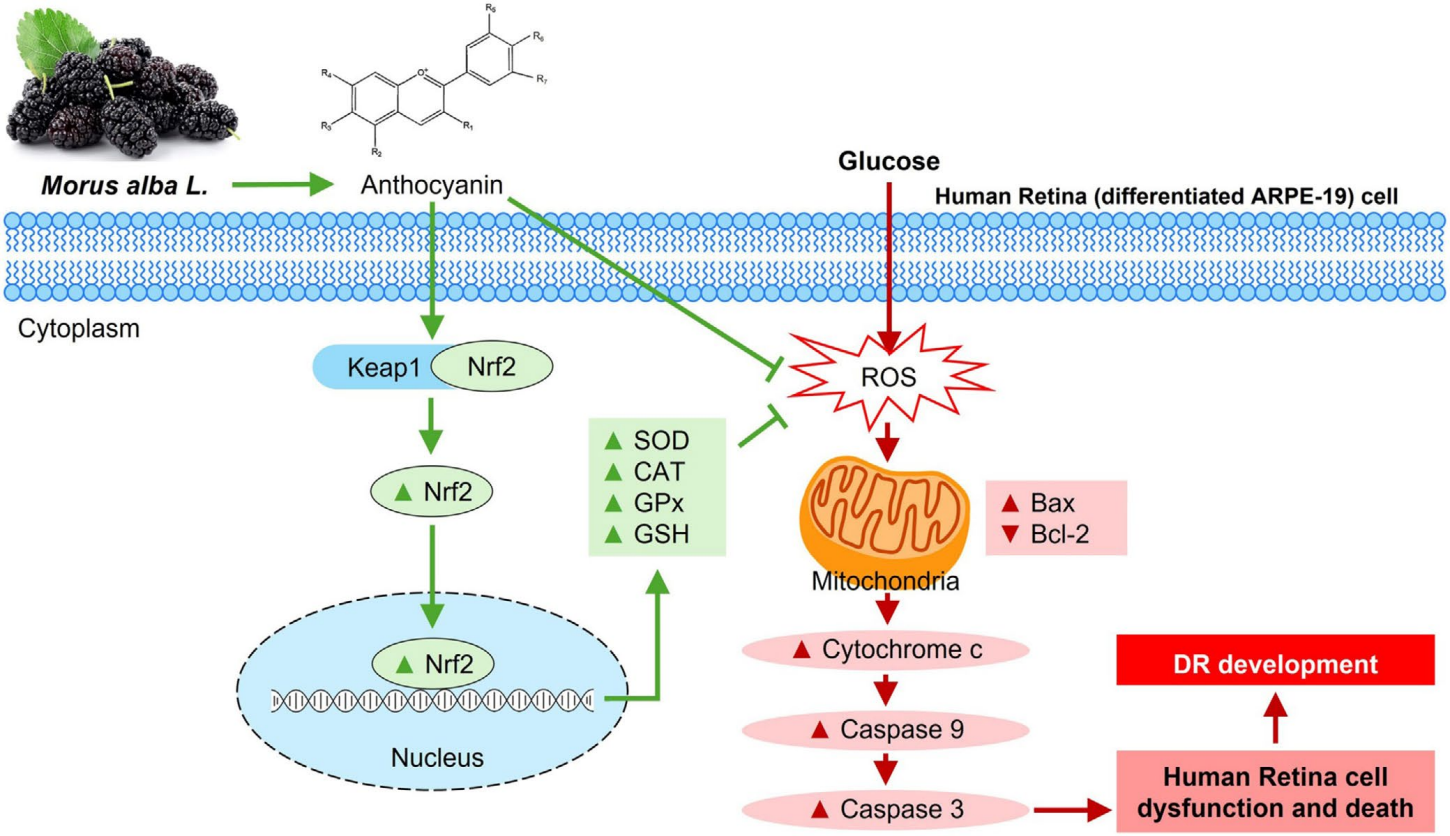
A schematic illustrates the protective effect of ME against glucose‐induced oxidative injury in differentiated ARPE‐19 cells. Pretreatment with ME protects differentiated ARPE‐19 cells by scavenging ROS and enhancing key enzymatic and nonenzymatic antioxidants by activating the key Nrf2 transcription factor.

These findings highlight that the antioxidant effects of ME are likely attributable to anthocyanins, particularly C3R and C3G. ME could protect human retinal cells from high glucose‐induced oxidative injury by reducing ROS levels, improving the crucial apoptotic proteins (Bax, cytochrome *c*, Bcl–2, and caspases–9 and –3), and enhancing the intracellular antioxidants (SOD, CAT, GPx, and GSH) via activating the essential transcription factor Nrf2 (Figure 11). However, as this study was conducted in vitro, further investigation is needed to assess the health benefits of mulberry, particularly with eye health, vision, and the prevention of diabetic retinopathy, using animal models and human studies.

## Author Contributions

**Pornpan Sukboon:** data curation (equal), formal analysis (equal), investigation (equal), writing – original draft (equal). **Rianthong Phumsuay:** formal analysis (equal), investigation (equal), methodology (equal). **Chadamas Promkum:** formal analysis (equal), investigation (supporting). **Parunya Thiyajai:** formal analysis (equal), investigation (equal), methodology (equal). **Monruedee Sukprasansap:** conceptualization (supporting), supervision (equal). **Chawanphat Muangnoi:** conceptualization (lead), data curation (equal), formal analysis (equal), funding acquisition (lead), methodology (equal), project administration (lead), writing – original draft (equal), writing – review and editing (equal).

## Conflicts of Interest

The authors declare no conflicts of interest.

## Data Availability

Data available on request from the authors.
